# An Integrated Map of HIV-Human Protein Complexes that Facilitate Viral Infection

**DOI:** 10.1371/journal.pone.0096687

**Published:** 2014-05-09

**Authors:** Dorothea Emig-Agius, Kevin Olivieri, Lars Pache, Hsin Ling Shih, Olga Pustovalova, Marina Bessarabova, John A. T. Young, Sumit K. Chanda, Trey Ideker

**Affiliations:** 1 Departments of Medicine and Bioengineering, University of California at San Diego, La Jolla, California, United States of America; 2 IP&Science, Thomson Reuters Scientific Inc., Carlsbad, California, United States of America; 3 Sanford-Burnham Medical Research Institute, La Jolla, California, United States of America; 4 The Salk Institute for Biological Studies, La Jolla, California, United States of America; George Mason University, United States of America

## Abstract

Recent proteomic and genetic studies have aimed to identify a complete network of interactions between HIV and human proteins and genes. This HIV-human interaction network provides invaluable information as to how HIV exploits the host machinery and can be used as a starting point for further functional analyses. We integrated this network with complementary datasets of protein function and interaction to nominate human protein complexes with likely roles in viral infection. Based on our approach we identified a global map of 40 HIV-human protein complexes with putative roles in HIV infection, some of which are involved in DNA replication and repair, transcription, translation, and cytoskeletal regulation. Targeted RNAi screens were used to validate several proteins and complexes for functional impact on viral infection. Thus, our HIV-human protein complex map provides a significant resource of potential HIV-host interactions for further study.

## Introduction

Human Immunodeficiency Virus (HIV) preferentially targets macrophages and CD4+T-cells of the immune system. Because of its small genome, encoding only 15 proteins and 3 polyproteins, HIV must interact extensively with the cellular machinery of the human host at all stages of its life cycle [1]. More than 20 drugs have been developed that target HIV proteins and act at distinct replication stages [2]. Given the frequency of drug resistance mutations in HIV, however, an emerging paradigm is to instead target host cell factors necessary for viral replication since these factors are relatively static [3]. Therefore, increasing our knowledge of these human host factors, their biological functions, and their interactions with HIV itself is of significant interest.

Toward this goal, three separate large-scale RNA interference (RNAi) screens have been aimed at the identification of so-called “HIV-dependency factors”, i.e. human proteins involved in HIV infection [4]–[6]. While only three genes were identified by all screens, substantially more overlap between screens was found at the level of functional clusters [7]. The limited overlap of genes from these studies is likely due to differences in the experimental approaches and implies that further analyses may reinforce some of these genes and also implicate new ones. Along these lines, Murali and colleagues demonstrated a powerful approach whereby all three RNAi screens were integrated with protein interaction networks to prioritize HIV-dependency factors based on their network proximity to other genes identified in the primary screens [8]. At the time, however, such efforts were limited by the available data on molecular interactions with HIV proteins, which had not yet been the focus of a systematic interaction mapping effort.

Very recently, Jaeger et al. sought to define the complete interaction network among HIV and human proteins in an attempt to better understand how HIV exploits the human cell machinery [9]. AP-MS (affinity–purified mass spectrometry) analysis was used to identify approximately 2,500 human proteins forming more than 10,000 physical interactions with HIV. Of these HIV-interacting human proteins, several were observed to be members of the same protein complex, suggesting that infection may be best understood by its effects at the modular level, not by effects on individual proteins. The AP-MS network provides a broad scaffold of HIV-human protein interactions on which to integrate complementary functional datasets.

Here, we integrate the Jaeger et al. network with complementary information on protein-protein interactions in humans and HIV-dependency factors to generate the first comprehensive map of HIV-human protein complexes. As in the earlier study by Murali et al., we employ the technique of network propagation, a powerful flow-based method to smooth information over a network. In the present study, network propagation is used to identify HIV-human complexes based on their significance for HIV proteins and their RNAi phenotypes. As has been shown in a study by Wuchty et al, known HIV dependency factors tend to directly interact with or be in close proximity to HIV proteins [10]. Therefore, efficient flow-based methods need to take prior knowledge into account about both HIV dependency factors and HIV-human interactions as accomplished here. Our study results in a global map of 40 HIV-human protein complexes involved in transcriptional control, translation, transport, and posttranslational modification, most of which have not yet been associated with HIV infection. Several of these complexes are explored through follow-up RNAi analysis which demonstrates the relevance of the identified HIV-human protein complexes.

## Results

### Scoring Human Proteins by Network Proximity

**Figure 1:**
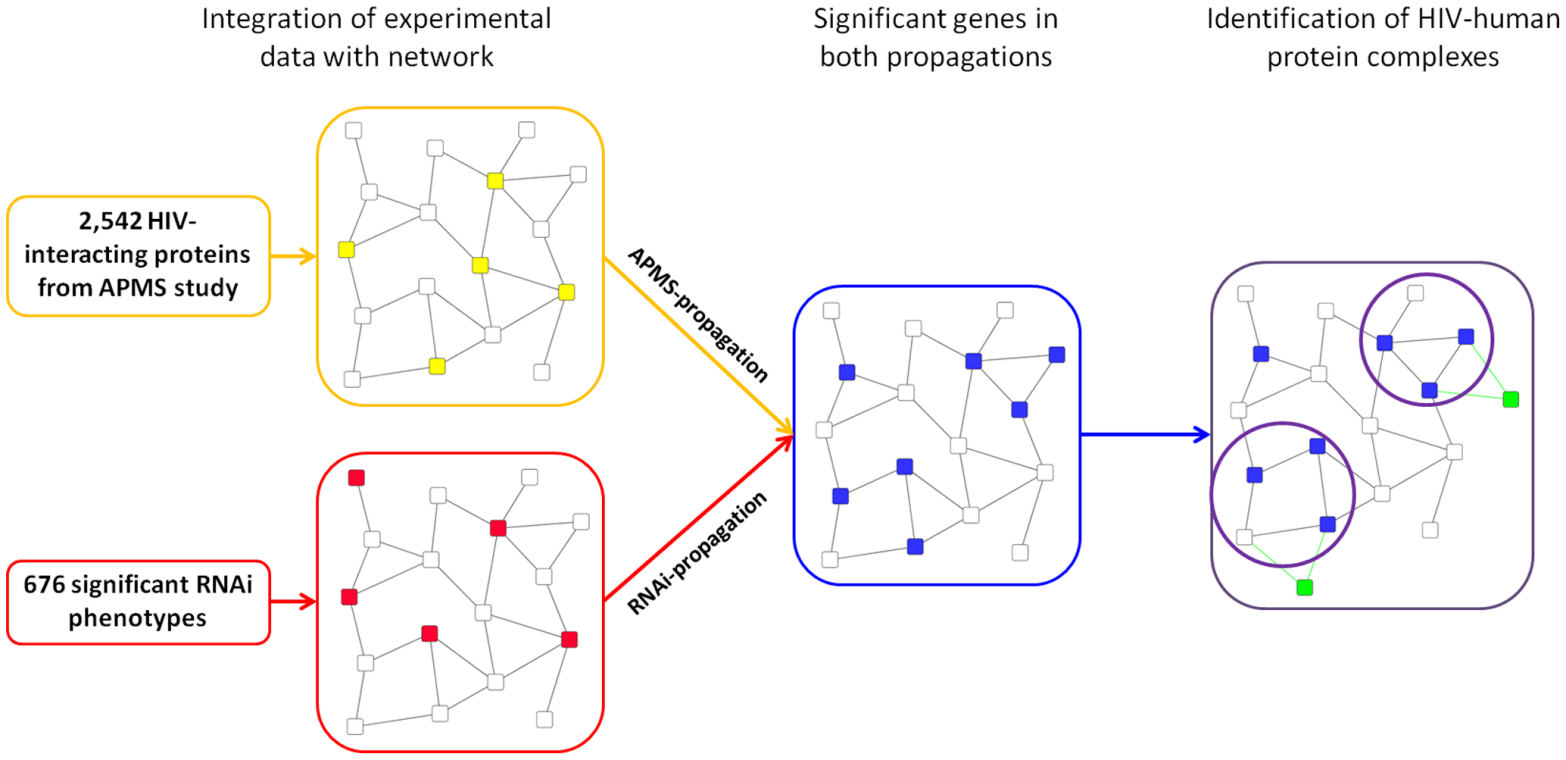
Workflow Overview. HIV-interacting proteins and RNAi phenotypes are mapped to a network of human protein functional interactions (yellow and red nodes respectively). Network propagation is performed separately for each of these two mappings. Significant genes are selected based on the combination of both propagation results (blue nodes). Finally, enriched HIV-human protein complexes are identified within the list of significant genes (HIV proteins added as green nodes, protein complexes highlighted by circles).

We first prioritized human proteins as HIV-dependency factors based on the integration of a human protein-protein interaction network with HIV-human protein interactions and, separately, RNAi phenotype data (Fig. 1).To provide a human protein-protein interaction network we used HumanNet [11] in which all gene pairs have been scored for their likelihood to participate in the same biological processes defined by the Gene Ontology database [12]. Thus, this network incorporates both physical and functional interactions between proteins instead of being restricted to physical protein interactions only. This allowed us to identify novel human proteins that are functionally related to known HIV-dependency factors but do not necessarily physically interact. HIV-human protein interactions were taken from Jaeger et al. and RNAi phenotypes from the three large-scale screens performed previously [4]–[6].

**Figure 2:**
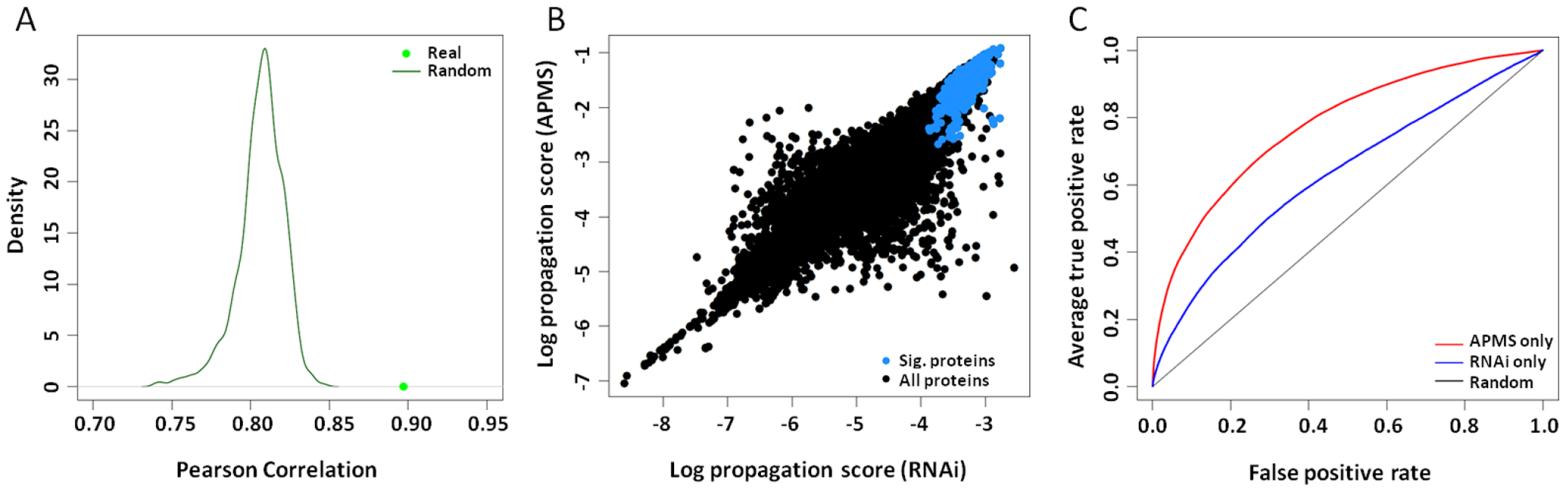
Predictive power and statistical results. (A) Pearson correlation of RNAi and APMS network propagation scores (green dot). The green line shows the density plot of random correlation coefficients based on permuting the relationship between network nodes and protein names. Note that random correlation is not zero due to the network structure of HumanNet which is not randomized. (B) The RNAi and APMS network propagation scores for each protein. Blue dots are proteins significant in both propagations. (C) ROC curve showing the predictive power of RNAi-propagation (blue) and APMS-propagation (red).

Each protein in HumanNet was scored with two types of information: [a] whether the protein was found by Jaeger et al. to interact with HIV in their APMS screen; and [b] whether the protein was associated with an RNAi phenotype. The technique of network propagation was then used to diffuse each of these scores, independently, over the HumanNet network (henceforth called APMS-propagation and RNAi-propagation respectively). The APMS- and RNAi-propagation scores were highly and significantly correlated, with a Pearson correlation of approximately 0.89 (Fig. 2A). Thus, genes with RNAi phenotypes in HIV infection are likely to be close to, or directly interacting with, HIV proteins.

At a significance level of p<0.0001 for both APMS-propagation and RNAi-propagation scores, we identified a final set of 554 human proteins with close proximity to HIV proteins as well as to human proteins with RNAi phenotypes, referred to as the *high-confidence RNAi-HIV set* hereafter (Fig. 2B, Table S1). Of these proteins, 382 were direct HIV interactors identified by AP-MS, 79 were identified directly by RNAi, and 148 were novel predictions identified by network proximity.

### Predictive Power of Network Analysis

To evaluate the predictive power of network propagation, we applied a two-fold cross-validation procedure, as follows. First, proteins with RNAi phenotype (Fig. 2C, RNAi only) or HIV-interacting proteins(Fig. 2C, APMS only) were divided in two equal-sized halves to create a training set and a test set. For each propagation (RNAi or APMS), we used the proteins in the training set as starting points for network propagation, resulting in a prioritized list of all proteins ranked by their scores. We then computed the sensitivity and specificity with which these scores predicted the proteins in the test set and rejected a set of randomly-chosen proteins, given a particular score threshold for prediction (Methods). As shown in Figure 2C, network propagation was able to recover these proteins with an Area Under ROC Curve (AUC) of 0.63 in the RNAi-propagation and an AUC of 0.77 in the APMS-propagation, which is well above the performance of a random predictor with expected AUC of 0.5.

To further validate our predictions experimentally, we arbitrarily selected 16 genes that were contained in the high-confidence RNAi-HIV set but had not been previously identified by RNAi screens. All of these 16 were tested by RNAi analysis (Table S2). For this assay, siRNA transfected 293T cells were infected with a VSV-G pseudotyped virus bearing a Fire Fly luciferase reporter. This vector does not express the HIV Envelope protein and is limited to a single cycle of infection. For 5 out of these 16 proteins (31%), gene knock-down resulted in at least a two-fold change of HIV infection, validating these proteins as HIV-dependency factors. By comparison, only 3 out of 54 randomly chosen genes (5%) led to at least two-fold change of HIV infection. Thus, network-based prediction of human HIV-dependency factors performs significantly better than random with a roughly 6∶1 enrichment ratio (p = 0.01, Fisher's Exact Test).

**Figure 3:**
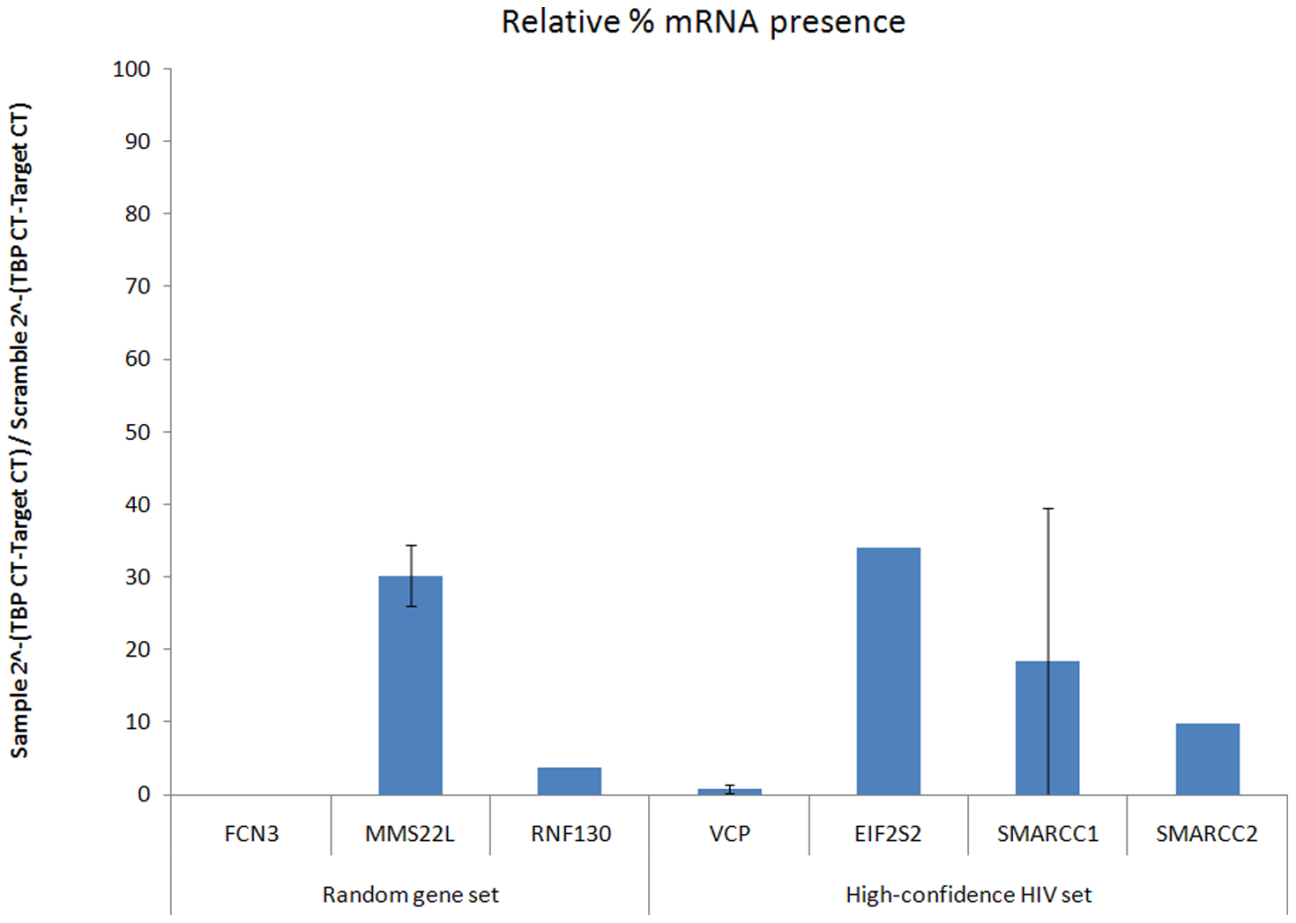
Validation of mRNA knock-down by siRNAs found to alter HIV infection. 293T cells were transfected with siRNA against the identified genes and two non-targeting scramble siRNAs. 72 h post-transfection, total RNA was harvested and used to make a cDNA library. The presence of the target gene and a housekeeping gene, TBP, was measured using QPCR. Target gene levels were normalized to TBP within in each sample. Values reported are normalized target gene levels compared to values observed in transfections with non-targeting scramble siRNAs.

To ensure that the observed HIV infectivity changes could be attributed to the knock-down of the target genes, we next validated the efficiency of our siRNA screening approach. 7 out of the 8 siRNAs which demonstrated at least a 2-fold effect on HIV infection were tested for their efficiency by which they reduced target gene mRNA levels. All 7 genes shown to alter HIV infection showed significant knock-down after siRNA transfection with at least 50% knock-down each (Fig. 3). FCN3 was undetectable following FCN3 siRNA transfection but was detectable in control samples (CT values of 35.181 and 34.525 for scrambles 1777 and 1776, respectively). Thus, our siRNA screening approach effectively reduced target gene mRNA levels and the previously described change in HIV infection can be attributed to the genes in question.

### A Map of HIV-Human Protein Complexes

**Figure 4:**
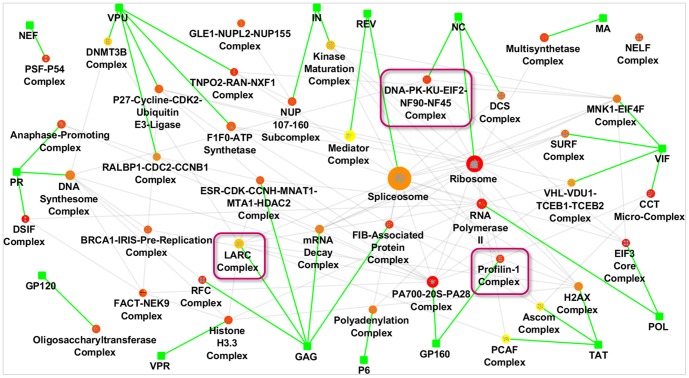
Map of HIV-human protein complexes. 40 identified human protein complexes are shown together with the HIV protein targeting the complex. Green rectangles correspond to HIV proteins. Human complexes are shown as ellipses. A color gradient from red (high) to yellow (low) indicates the average rank of the complex in the APMS- and RNAi-propagations. Node size corresponds to number of subunits in the complex. Gray edges represent functional interactions between the human complexes; green edges are HIV-human interactions. Purple boxes indicate protein complexes that were selected for follow-up RNAi screens.

The prioritization of human HIV-dependency factors is an important first step towards understanding how HIV exploits the human cellular machinery. However, since proteins often perform their functions in concert, i.e. through the formation of protein complexes, we sought to analyze HIV-dependency factors at the complex level. Starting with the high-confidence RNAi-HIV set of 554 proteins resulting from the previously-described network propagation, we found that these proteins were significantly enriched for 40 protein complexes from the CORUM database [13]. Of these 40 complexes, 27 had not been reported in previous studies of HIV, while the remaining 13 complexes had been identified by at least one previously published analysis (Table 1). To create an HIV-human protein complex map, we assigned the interacting HIV protein with the highest interaction confidence to each complex (Fig. 4, Methods). In this map, 15 of 18 HIV proteins directly target human complexes and36 of the 40 complexes in the map have at least one subunit that directly interacts with HIV.

**Table 1:** Comparison of results from different studies.

Complex Name	Function	This study	Jaeger	Murali	Bushman
**ASCOM complex**	Transcription Activation, DNA Topological Change	1	-	-	-
**BRCA1-IRIS-pre-replication complex**	DNA Replication	1	-	-	-
**DCS complex**	Regulation of RNA Splicing	1	-	-	-
**DNA-PK-Ku-eIF2-NF90-NF45 complex**	Phosphorylation, DNA Repair	1	-	-	-
**DNMT3B complex**	DNA Topological Change, DNAMethylation	1	-	-	-
**DSIF complex (DRB sensitivity-inducing factor complex)**	Transcription Repressor, RNA Elongation	1	-	-	-
**ESR1-CDK7-CCNH-MNAT1-MTA1-HDAC2 complex**	Transcription, Phosphorylation	1	-	-	-
**FACT-NEK9 complex**	DNA Topological Change, Phosphorylation, RNA Elongation	1	-	-	-
**FIB-associated protein complex**	rRNA Processing	1	-	-	-
**GLE1-NUPL2-NUP155 complex**	Protein Transport	1	-	-	-
**H2AX complex II**	DNA Topological Change, DNA Repair, RNA Elongation	1	-	-	-
**Histone H3.3 complex**	Protein Complex Assembly	1	-	-	-
**Kinase maturation complex 1**	Kinase Signaling	1	-	-	-
**LARC complex (LCR-associated remodeling complex)**	Transcription, Acetylation, DNA Topological Change	1	-	-	-
**MNK1-eIF4F complex**	Translation Initiation	1	-	-	-
**mRNA decay complex**	RNA Degradation	1	-	-	-
**NELF complex (Negative elongation factor complex)**	Transcription Repressor, RNA Elongation	1	-	-	-
**Oligosaccharyltransferase complex**	Glycosylation	1	-	-	-
**PCAF complex**	Transcription, DNA Topological Change, Acetylation	1	-	-	-
**Polyadenylation complex**	RNA 3′-end processing	1	-	-	-
**Profilin 1 complex**	Endocytosis, Complex Assembly, Actin Skeleton	1	-	-	-
**PSF-p54(nrb) complex**	DNA Repair	1	-	-	-
**RalBP1-CDC2-CCNB1 complex**	Phosphorylation	1	-	-	-
**RFC complex (activator A 1 complex)**	DNA Replication, Phosphate Metabolism	1	-	-	-
**SMG-1-Upf1-eRF1-eRF3 complex (SURF)**	RNA Transport, Phosphorylation, mRNAStability	1	-	-	-
**TNPO2-RAN-NXF1 complex**	RNA Transport	1	-	-	-
**VHL-VDU1-TCEB1-TCEB2 complex**	Proteasomal Ubiquitin-Dependent Protein Catabolism	1	-	-	-
*PA700-20S-PA28 complex*	Proteasomal Ubiquitin-Dependent Protein Catabolism	1	1	1	1
*Multisynthetase complex*	tRNA Ligase Activity	1	1	-	1
*DNA synthesome complex*	DNA Replication	1	1	-	-
*p27-cyclinE-Cdk2 - Ubiquitin E3 ligase complex*	Ubiquitination, Phosphorylation	1	1	-	-
*EIF3 core complex*	Translation Initiation	1	1	1	-
*F1F0-ATP synthase, mitochondrial*	Energy Generation	1	1	1	-
*Anaphase-promoting complex*	Ubiquitination, Cell Cycle Control	1	-	1	-
*Nup 107-160 subcomplex*	Protein Transport	1	-	1	-
*Ribosome, cytoplasmic*	Protein Biosynthesis	1	-	1	-
*Mediator complex*	Transcription Activation	1	-	1	1
*Spliceosome*	RNA Splicing	1	-	1	1
*CCT micro-complex*	Protein Folding	1	-	-	1
*RNA polymerase II holoenzyme complex*	Transcription	1	-	-	1
P-TEFb complex	Transcription, RNA elongation	-	1	-	1
MCM complex	DNA Replication	-	1	-	-
COP9 signalosome	Transcription, Signal Transduction	-	1	-	-
SMN complex	RNA Splicing, Protein Complex Assembly	-	1	-	-
HDAC3/NCOR complex	Transcription Repressor, Acetylation	-	1	-	-
Dynein complex	Transport	-	1	-	-
Profilin 2 complex	Endocytosis, Complex Assembly, Actin Skeleton	-	-	-	1
SNW1 complex	RNA Splicing	-	-	-	1
MHC protein complex	Immune Response	-	-	1	-
Respiratory chain complex I	Energy Generation	-	-	1	-

The table lists all protein complexes identified by our method, as well as the complexes identified in three previous analyses from Jaeger et al, Murali et al, and Bushman et al. Bold complexes correspond to those uniquely identified in our study, italic to those identified by us and by at least one previous study. The remainder corresponds to protein complexes identified in previous analyses only.

### Validation and Further Analysis of Selected Protein Complexes

We selected three protein complexes which had not been identified in previous analyses for further investigation as novel functional candidates for HIV infection. These were the Profilin-1 complex, which is involved in regulation of the cytoskeleton; the DNA-PK-Ku-eIF2-NF90-NF45 complex, which is involved in DNA repair and regulation of viral transcription; and the LARC complex, which is involved in chromatin remodeling (Table S3). None of the subunits of Profilin-1 and DNA-PK-Ku-eIF2-NF90-NF45 had been identified in previous RNAi screens, and thus none were contained in the initial set of proteins for the RNAi propagation. In case of the LARC complex, 2 of the 19 subunits were identified in previous RNAi screens, although the LARC complex itself was not implicated in these analyses.

#### Profilin-1 Complex

**Figure 5:**
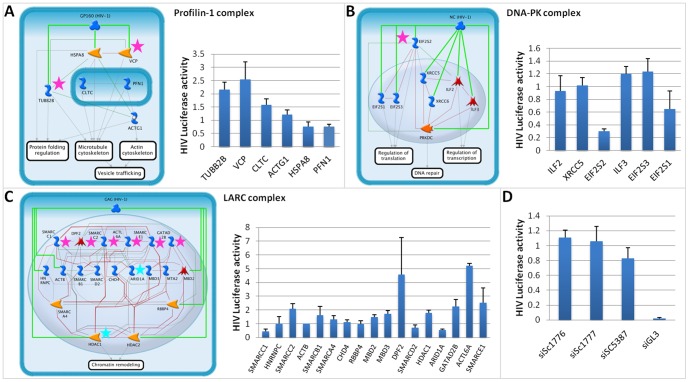
Selected complexes and RNAi screening results. (A) Profilin-1 complex interacting with GP160. (B) DNA-PK-Ku-eIF2-NF90-NF20 complex interacting with NC. (C) LARC complex interacting with Gag. Interactions within the complex represent functional interactions from HumanNet (green), manually curated interactions from the Metabase resource (gray) or from both sources (red). Pink vs. turquoise stars correspond to proteins that were confirmed in our RNAi validation screen vs. previous screens, respectively. Orange nodes are kinases, red transcription factors, blue are binding proteins as classified in Metabase. The bar plots show the HIV luciferase activity of the sample normalized by the HIV luciferase activity of control siRNAs. (D) HIV luciferase activity for three non-targeting siRNAs (positive controls) and luciferase-targeting siGL3 (negative control) performed simultaneously with siRNA transfections shown in A, B, and C.

The Profilin-1complex contributes to actin polymerization and endocytosis [14]. Inhibition of Profilin-1-mediated actin filament formation was found to inhibit HIV-1 replication [15]. However, none of the complex subunits have been reported in published RNAi screens. In our study, 5 of the 6 Profilin-1 complex components were predicted to be HIV-dependency factors by network propagation. We thus re-screened all six subunits using siRNA and were able to validate *TUBB2B* and *VCP* as HIV-dependency factors (Fig. 5A). Based on the AP-MS network analysis, *GP160* is expected to interact with the complex. *VCP* has previously been shown to play a role in Vpu-dependent CD4 degradation [16], but it is likely to impact the VSV-G pseudotyped HIV single-cycle infection used here through a separate mechanism.

The DNA-PK-Ku-eIF2-NF90-NF45 complex is a DNA-binding complex involved in DNA repair [17]. This complex consists of eight subunits: 3 translation initiation factors, 2 interleukin-enhancing binding factors, and 3 DNA-binding subunits. Based on the AP-MS network analysis, *NC* is likely to interact with the complex. Three of the 8 subunits (*ILF2*, *XRCC5*, *EIF2S2*) were predicted as HIV-dependency factors by network propagation. Experimental rescreening of the subunits in this complex indicated that *EIF2S2* is the important subunit for infection (Fig. 5B). Conflicting prior data exist as to whether or not *DNA-PK* and *Ku* are important for infection [18], [19]. The controversy can be explained in part by differences in the experimental approaches and our findings suggest that *EIF2S2*impacts HIV replication independent of DNA-*PK* and *Ku*. Understanding regulation of *EIF2S2* function by the DNA-PK-Ku-eIF2-NF90-NF45 complex may help define parameters where DNA-repair affects HIV infection.

The LARC complex is composed of 19 different subunits, 4 of which are predicted to be HIV-dependency factors by network propagation. The complex plays an important role in chromatin remodeling which is important for transcriptional regulation [20] and methylation-dependent gene silencing [21]. Two subunits, *HDAC1* and *ARID1A*, were found to be HIV-dependency factors in previous RNAi screens, and recent publications have identified *ACTL6A*, *DPF2* and *SMARCC2* as regulators of HIV-1 transcription [22], [23]. siRNA knock-down of the 4 predicted subunits revealed significant effects of 2 subunits on HIV infection, namely *SMARCC1* and *SMARCC2* (Fig. 5C). Furthermore, knock-down of the non-predicted subunits revealed significant effects of *GAGD2B* and *SMARCE1* on HIV infection and confirmed recent implications of *DPF2* and *ATCL6A* as HIV-dependency factors. These results suggest that the LARC complex as a whole may play a role in HIV transcription.

## Discussion

Understanding how HIV exploits the human cellular machinery is essential for developing novel strategies for long term treatment. Previous large-scale studies have mainly focused on the identification of single proteins that serve as HIV-dependency factors and on the characterization of general biological processes they are involved in.

In this analysis, we provide the first global map of human protein complexes involved in HIV infection. Integrating proteomic and genetic datasets allowed us to reveal additional regulatory components of protein complexes that are already associated with regulation of HIV-1 replication as well as currently unknown protein complexes that play a role in HIV infection. Validation of several predicted protein complexes with targeted RNAi screening demonstrate the high value of the human-HIV protein complex map for future studies (Fig. 4).

Our map not only provides a resource for tracking targeted protein complexes at different stages of the HIV life cycle, but it also provides testable hypotheses of potentially hijacked complexes by HIV for follow-up experiments. Ultimately, our map may lead to the identification of druggable human protein complexes and allow for a shift in therapeutic strategies towards human targets.

## Methods

### Network Data

The human protein interaction network employed in this study is HumanNet, which comprises 476,399 functional interactions among a total of 16,234 human genes [11]. HumanNet links proteins based on a combination of 21 diverse types of biological evidence including genetic and protein interactions, co-expression and co-citation data. Notably, interactions in HumanNet do not necessarily correspond to physical interactions between human gene products but represent close functional relationships between the genes. The interactions are predicted using a supervised approach that learns what combination and weighting of evidence types best predicts interactions between pairs of genes with the same Gene Ontology (GO) Biological Process annotations. This scheme is then used to compute the likelihood of all protein pairs based on the combination and weight of available evidence.

Additionally, we retrieved 11,432 interactions between HIV and human proteins from a recently published AP-MS study [9]. Each interaction in this dataset is annotated with a confidence score (MiST score) describing its specificity, reproducibility, and abundance. The HIV-human network contains interactions between 18 HIV proteins and polyproteins and 2,635 human proteins, of which 2,542 also have high-scoring interactions in HumanNet.

### RNAi Screening Data

We obtained a list of 749 human genes for which RNAi knockdown had a significant effect on HIV infection, based on the union of the results of three publicly available RNA interference screens [4]–[6]. As noticed previously, the overlap between the genes reported in the three studies is small with three genes identified by all and 26 genes identified by any two screens [3]. Of the 749 genes, 676 could be mapped to proteins in HumanNet and were therefore used in our study.

### Network Propagation

The input to network propagation is an undirected graph *G* = (*V,E*), where *V* is the set of nodes and *E* is the set of edges. In each iteration of the algorithm the node scores are updated using the following formula:
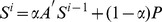
where *S^i^* and *S^i-1^* represent vectors of node scores in iterations *i* and *i-1*. *A′* is the adjacency matrix of graph *G*, where each entry is normalized by the degrees of the respective nodes. *P* corresponds to a vector encoding prior knowledge, i.e. the scores assigned to the starting points. Finally, alpha is a weighting factor, assigning the probability for propagating the flow through the network and the restart probability at the starting points.

We applied network propagation separately for RNAi phenotypes (*RNAi-propagation*) and HIV-interacting proteins (*APMS-propagation*). In both cases, we used HumanNet as the input network and alpha  = 0.8, which provided the most accurate results in a previous study [8]. In the RNAi-propagation, we assigned initial scores to proteins based on their pattern of detection in the three RNAi screens: Genes detected in all screens received *P* = 1; those detected in two screens *P* = 2/3, those detected in one screen *P* = 1/3, and *P* = 0 otherwise. In the APMS-propagation, the HIV-interacting human proteins were used as starting points. For each human protein in the network, we extracted all of its HIV interactions and assigned it the maximum MiST score of these interactions as initial scores.

### Selection of the High-Confidence RNAi-HIV Set

The high-confidence RNAi-HIV set was selected as the most significant proteins in the RNAi- and the APMS-propagation using protein-centered statistical models. The idea of a protein-centered model is to determine the significance of a protein score compared to the expected score for that protein instead of defining a global score cutoff for all proteins in the network. This was achieved by comparing the protein score to the underlying null model for the respective node. We chose a conservative random network model, in which we permuted the node labels while the network structure itself was preserved. To obtain accurate p-values even for the tails of the null distributions, we made use of a tail approximation introduced in a study by Knijnenburg and colleagues [24]. As demonstrated, tail distributions can be modeled by a generalized Pareto distribution and result in accurate p-values even if the number of permutations is small. The significance cutoff for the FDR-corrected p-values was set to 0.0001 and all genes with a significant p-value in both the RNAi- and the APMS-propagation were selected for the high-confidence RNAi-HIV set.

### Predictive Power

We assessed the predictive power of the RNAi-propagation and the APMS-propagation separately, using the same procedure. For each propagation we performed 100 simulations, in which we randomly divided the respective set of starting points (human proteins with RNAi phenotypes or human proteins with HIV interactions) into two equally-sized halves. Network propagation was performed using the first half as starting points, and the second half was then used to evaluate the predictive power of the propagation based on their ranks within the propagation results. We assessed the predictive power using a receiver-operating-curve (ROC) plot generated with the ROCR R-package [25]. It should be noted that we considered all genes that were not in the starting lists of the RNAi- or APMS-propagation to be false positives, even though some of them may be as yet unknown host factors. The specificity of our method may thus be higher in reality such that the ROC curve can be seen as a lower bound on the predictive power.

### Identification of HIV-Human Protein Complexes

Using the list of predicted genes, we identified significantly enriched protein complexes using a hypergeometric test. We obtained manually curated protein complexes from the CORUM database [26], downloaded in January 2012. For the hypergeometric test, we mapped the complexes onto HumanNet and only considered those genes within the complexes that were also contained in HumanNet. We kept all protein complexes with an FDR-corrected p-value below 0.05. We excluded overlapping protein complexes by first ordering the significant protein complexes by the number of predicted genes within them. We then selected the complexes iteratively based on this ranking, excluding complexes that shared proteins with those that had been previously selected.

Next, for each complex we identified the HIV protein most likely to target it, considering those HIV-human protein interactions with MiST score >0.2. For each HIV protein, we summed the MiST scores of all interactions between the HIV protein and complex subunits. The HIV protein with the highest sum of MiST scores was assigned to the human protein complex.

### siRNA validation: Viral production

VSV-G pseudotyped HIV-1 bearing a Fire Fly Luciferase (VSV-G HIV LUC) was produced by the Viral Vector Core, Salk Insitute, La Jolla, CA, using PEI transfection of 293T cells [6]. Infectious supernatants were collected 48 h post-transfection, cleared of cellular debris by centrifugation and then filtration. Infectious stocks were DNase-treated before use.

### siRNA validation: Transfection and HIV infectivity assay

Confluent 293 T cells were trypsinized and resuspended in 20% FBS OptiMEM at 1.5×105 per ml. 0.5 pg of Dharmacon Smart Pool siRNA was mixed with 10 µl of OptiMEM. 45 nl of RNAiMax was added to 10 µl OptiMEM. Both mixtures were incubated for 5 minutes, then combined. After 20 minute incubation at room temperature, the mixture was added to 20 µl media containing 3000 cells in a 384-well white walled plate. After 48 h incubation at 37°C, 0.3125 µl (MOI = 0.05) of VSV-G pseudotyped HIV-1 LUC in 10 µl 10% FBS DMEM was added and cells were incubated for an additional 24 h. Luciferase activity was determined after addition of 25 µl Brite Glo reagent (Promega). Uninfected replicate wells were maintained for each transfection and assayed for cell viability with 25 µl of ATP Lite (Perkin-Elmer) at 72 h post-transfection. Luminescence was measured on an Enspire luminometer (Perkin-Elmer).

### siRNA validation: Efficiency of siRNA-mediated mRNA knock-down

siRNA for each target or two scrambles (1776 and 1777) were diluted to 50 nM in OptiMEM (Life Technologies, Carlsbad, CA). RNAiMax (Life Technologies, Carlsbad, CA) was diluted 3∶500 separately in OptiMEM and added to an equal volume of the siRNA dilution. siRNA/lipid mixtures were incubated for 30 minutes at room temperature. 293 T cells were lifted with Trypsin/EDTA, pelleted, and resuspended in 20% FBS DMEM with no antibiotics. An equal volume containing 10,000 cells was added to siRNA/lipid mixtures and incubated for 3 days at 37°C in a humidified incubator with 5% CO2. Total RNA was extracted from cells using Machery-Nagel 96-well total RNA vacuum kit. RNA was pelleted with glycogen, EtOH, and potassium acetate, washed with 70% EtOH, and resuspended in 5 µl water. 3.5 µl of RNA was added to 0.5 µl of iSCRIPT Reverse Transcriptase and 1 µl of 5X iSCRIPT buffer (Bio-Rad, Hercules, CA). cDNA synthesis was performed at 42°C for 30 minutes. RT was inactivated by incubation at 85°C. The cDNA product was diluted 1∶3 in dH20. QPCR was performed with primers designed by the Harvard Primer Bank (http://pga.mgh.harvard.edu/primerbank/). Primers were used at 0.5 um with 2 µl of diluted cDNA product and iTaq Universal Sybr Green master mix (Bio-Rad, Hercules, CA). QPCR amplification was performed in an Applied Biosystems Viia 7 thermocycler. Melt curve analysis was performed to determine single fragment production. For each siRNA transfection, the target gene and a housekeeping gene, TBP, were amplified in separate reactions. The threshold cycle (CT) was determined for each target and TBP. The amount of target gene was related to TBP amounts by the following formula 2∧-(TBP CT – Target CT). The relative level of each gene was then normalized to the average of the two scrambles and then multiplied by 100%.

## Supporting Information

Table S1 High-confidence RNAi-HIV set. The list of 554 genes that were predicted in both RNAi- and APMS- propagation.
(XLSX)

Table S2 Genes selected for validation. Sheet 1 shows the 16 genes that were randomly drawn from the high-confidence set together with their RNAi result. Sheet 2 shows the list of genes randomly selected from the human genome together with their RNAi results.
(XLSX)

Table S3 Protein complexes involved in HIV infection. The list of predicted Corum protein complexes together with their protein subunits and biological functions. Colored complexes have been validated in follow-up RNAi experiments.
(XLSX)
